# Exploring myometrial microenvironment changes at the single-cell level from nonpregnant to term pregnant states

**DOI:** 10.1152/physiolgenomics.00067.2023

**Published:** 2023-11-13

**Authors:** Kaiyuan Ji, Junmin Zhong, Long Cui, Xiaodi Wang, Li-Na Chen, Bolun Wen, Fan Yang, Wenfeng Deng, Xiuyu Pan, Lele Wang, Junjie Bao, YunShan Chen, Huishu Liu

**Affiliations:** Guangzhou Key Laboratory of Maternal-Fetal Medicine, Guangzhou Women and Children’s Medical Center, Guangzhou Medical University, Guangzhou, People’s Republic of China

**Keywords:** myometrium, pregnancy, single-cell RNA sequencing, spatial transcriptomics

## Abstract

The microenvironment and cell populations within the myometrium play crucial roles in maintaining uterine structural integrity and protecting the fetus during pregnancy. However, the specific changes occurring at the single-cell level in the human myometrium between nonpregnant (NP) and term pregnant (TP) states remain unexplored. In this study, we used single-cell RNA sequencing (scRNA-Seq) and spatial transcriptomics (ST) to construct a transcriptomic atlas of individual cells in the myometrium of NP and TP women. Integrated analysis of scRNA-Seq and ST data revealed spatially distinct transcriptional characteristics and examined cell-to-cell communication patterns based on ligand-receptor interactions. We identified and categorized 87,845 high-quality individual cells into 12 populations from scRNA-Seq data of 12 human myometrium tissues. Our findings demonstrated alterations in the proportions of five subpopulations of smooth muscle cells in TP. Moreover, an increase in monocytic cells, particularly M2 macrophages, was observed in TP myometrium samples, suggesting their involvement in the anti-inflammatory response. This study provides unprecedented single-cell resolution of the NP and TP myometrium, offering new insights into myometrial remodeling during pregnancy.

**NEW & NOTEWORTHY** Using single-cell RNA sequencing and spatial transcriptomics, the myometrium was examined at the single-cell level during pregnancy. We identified spatially distinct cell populations and observed alterations in smooth muscle cells and increased M2 macrophages in term pregnant women. These findings offer unprecedented insights into myometrial remodeling and the anti-inflammatory response during pregnancy. The study advances our understanding of pregnancy-related myometrial changes.

## Graphical abstract

**Figure:**
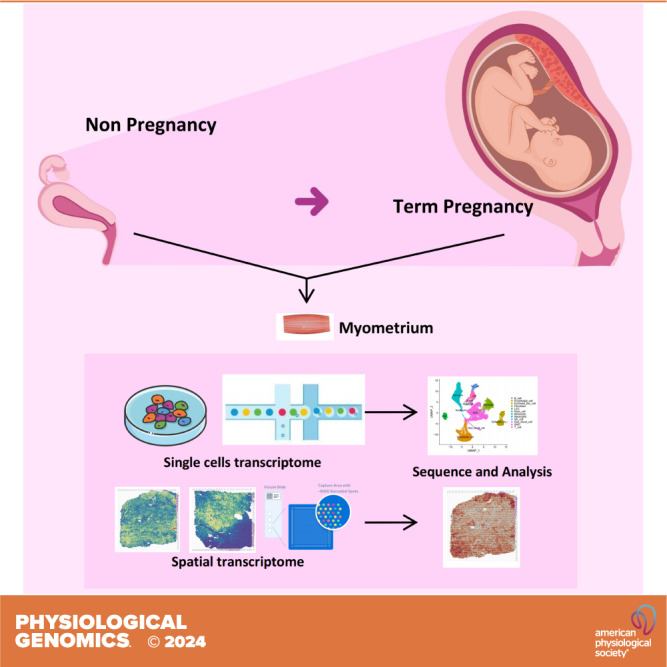

## INTRODUCTION

The myometrium is the muscular component of the uterus and serves functions such as supporting fetal growth and development, facilitating smooth childbirth, maintaining uterine stability, and providing oxygen and nutrients to meet the fetus’s needs (1, 2). From conception to the term of pregnancy, the length, weight, and capacity of the uterus increase from ∼7 to 20 cm and 70 g to over 1,000 g and 25 to 5,000 mL, respectively (3). Preterm birth, dystocia, adenomyosis, and leiomyoma can all be attributed to disorders affecting the myometrium, significantly impacting the health of both mothers and neonates (1, 4). Globally, ∼15 million neonates are born prematurely each year, with over 1 million succumbing to complications related to prematurity (5, 6). There is a lack of effective treatments to prevent preterm birth (7, 8). Our understanding of the physiological alterations occurring in the myometrium during pregnancy remains limited. Therefore, there is an urgent need for research focused on investigating changes in cell populations and molecular mechanisms within the myometrium.

Progesterone and estrogen are responsible for the thickening of the myometrium in the early stages of pregnancy (9, 10). Twelve weeks after conception, the myometrium exhibits hypertrophy and elongation owing to the growth of the fetus and the placenta (3). Smooth muscle cells (SMCs) are the predominant cell population in the myometrium, and their abundance and size increase during pregnancy (1). Previous studies have described two distinct phases in SMC growth during pregnancy, i.e., the proliferative phase (during which antiapoptotic proteins increase) and the synthetic phase (SMC size increases) (11, 12), which is mainly supported by the mechanical stretching and progesterone (11, 13, 14). An increase in cellular protein and significant changes in the mass and composition of the extracellular matrix (ECM) are characteristic of cellular hypertrophy (12). Quiescence of SMCs is promoted by progesterone, which suppresses prostaglandin- and oxytocin-dependent inflammatory responses and contractile activities before labor (15).

Normally, the fetus grows and develops within the mother without significant immune suppression, despite being a semiallogeneic graft (16–18). Previous studies on immunological alterations at the maternal/fetal interface have indicated that a balance of immune cell constituents was essential for fetomaternal tolerance (19–21). During pregnancy, adaptive immune cells are involved in maintaining fetomaternal tolerance., and malfunction of these cells leads to labor at or before term (22). Above all, macrophages play a critical homeostatic regulatory role in term pregnancy and are implicated in spontaneous preterm birth and fetal inflammatory injury (23). In term pregnancy, circulating maternal leukocytes (innate and adaptive) are recruited into the myometrium and to the decidual tissues through chemotactic processes (23). Regulatory T cells are maintained throughout pregnancy to induce tolerance to fetal antigens and allow conceptus implantation and maintenance until late gestation (24–26). However, the panorama of immune cells and their alterations during pregnancy remains unknown.

The myometrium undergoes drastic changes with regard to the transcriptome, epigenome, and proteome (27). Bulk RNA sequencing (RNA-Seq) of human myometrial tissues from nonpregnant (NP) and term pregnant (TP) women indicated an increase in the metabolic, inflammatory, and PDGF signaling pathways (27). Accessible genome of myometrium changed and the enrichment of binding motifs on changed regions are hormone and muscle regulators such as progesterone receptor and Krüppel-like factors (27). Single-cell RNA-Seq (scRNA-Seq) is currently developing rapidly, and it facilitates specific profiling of cell populations and gene expression at the single-cell level (28, 29). The single-cell atlas of the human myometrium during spontaneous term labor indicated that major cell populations were SMCs, monocytes/macrophages, fibroblasts, and endothelial cells (28, 30). scRNA-Seq of the normal human myometrium has identified SMCs, fibroblasts, natural killer (NK), T cells, B cells, myeloid cells, and endothelial cells (31). However, to our knowledge, a study integrating high-throughput scRNA-Seq and spatial transcriptomics (ST) for exploring cell populations of the myometrium during pregnancy is still absent.

Our study aimed to provide a comprehensive cell atlas of the transcriptome for myometrium cell populations in NP and TP samples using scRNA-Seq. We also used ST to visualize cell population and gene expression information in spatial organization (32, 33). These findings will help establish a more thorough understanding of myometrial remodeling during gestation and provide a basis for such research on preterm birth.

## MATERIALS AND METHODS

### Sample Collection

Myometrial tissue of NP samples was collected from the normal myometrium (excluded both the uterine serosa and endometrium, at least 3 cm from the nearest leiomyoma) of NP unmenopausal women with hysterectomy or myomectomy surgery for hysteromyoma as previous research (27). Myometrial tissue of TP samples was collected from the lower segment of women with single pregnant full-term woman who underwent cesarean section, with no pregnancy complications or placenta previa. All individuals had no regular contractions and progressive cervical dilation and were of reproductive age (30, 34). All tissues were obtained at Guangzhou Women and Children’s Medical Center (Guangzhou, PR China). The clinical details of patients gathered by clinical phenotype are provided in Supplemental Table S1. This research was approved by the Ethics Committee of Guangzhou Women and Children Medical Center (Nos. 201915401 and 2018041701), and each patient signed informed consent for this study.

### scRNA-Seq and Processing

Single-cell suspensions were loaded to 10× chromium to capture 10,000 single cells according to the manufacturer’s instructions of the 10X Genomics Chromium Single-Cell 3′ kit (V3). The following cDNA amplification and library construction steps were performed according to the standard protocol. Libraries were sequenced on an Illumina NovaSeq 6000 sequencing system (paired-end multiplexing run, 150 bp) by LC-Bio Technology (Hang Zhou, PR China) at a minimum depth of 20,000 reads per cell.

### scRNA-Seq Data Processing

Sequencing results were demultiplexed and converted to FASTQ format using Illumina bcl2fastq software (version 2.20). Sample demultiplexing, barcode processing, and single-cell 3′ gene counting used the Cell Ranger pipeline (https://support.10xgenomics.com/single-cell-gene-expression/software/pipelines/latest/what-is-cell-ranger), and scRNA-Seq data were aligned to the Ensembl genome GRCh38 reference genome. The Cell Ranger output was loaded into Seurat (version 4.0.2) to be used for dimensional reduction, clustering, and analysis of scRNA-Seq data (35). Quality control threshold was as follows: all genes expressed in less than three cells were removed, number of genes expressed per cell >200 as low and <6,000 as high cutoff, and percentage of mitochondrial DNA-derived gene expression was < 25%. The published scRNA-Seq data of five normal NP myometriums and three TP myometriums were downloaded from the Gene Expression Omnibus (GEO) database (GSE162122) and Genome Sequence Archive (GSA) database (HRA002852) (30, 36). Doublets were discarded using DoubletFinder (version 2.0.3) with the default parameters (37). To visualize the data, we further reduced the dimensionality of all cells using Seurat and used Unsupervised Uniform Manifold Approximation and Projection (UMAP) to project the cells into two-dimensional space. The data were then normalized by “SCTransform” with Seurat (38). This functioned as an alternative to the NormalizeData, FindVariableFeatures, and ScaleData workflow. All data from 10 samples were harmonized and integrated using the “FindIntegrationAnchors” and “IntegrateData” functions in Seurat (35). Principal component analysis was performed to reduce the dimensionality of the data. To find clusters, a *K*-nearest neighbor graph was constructed by “FindNeighbors” and cells were grouped together based on the top 30 principal components by the “FindClusters” function in Seurat. Marker genes for each cluster were identified with the “MAST” algorithm with default parameters via the FindAllMarkers function in Seurat. Subclustering for SMCs and immune cells was performed in the same way. Differentially expressed genes (DEGs) of each cell population between groups were calculated using the pseudobulk_method of DESeq2 in the Libra package (39). Cell-cell communication networks were identified and visualized by the CellChat (version 1.1.2) package with standard workflow (40). Pseudotime analyses were performed in Monocle (version 2.22.0) (41). The “dispersionTable” function was used to identify highly variable genes. Trajectories were constructed with the Discriminative Dimensionality Reduction with Trees (DDRTree) method.

### ST Library and Sequencing

Tissues of the myometrium were harvested, cut into ∼10-mm-thick tissue blocks, and embedded and frozen in optimal cutting temperature (OCT) compound using liquid nitrogen. Myometrium blocks were cryosectioned at a thickness of 10 μm followed by staining with hematoxylin and eosin. Visium spatial gene expression was processed using a 10X Visium spatial gene expression slide and reagent kit (PN-1000184, 10X Genomics). For prepermeabilization, sections were incubated at 37°C with 70 μL permeabilization enzyme for 20 min. Each well was washed with 100 μL saline sodium citrate, and 75 μL reverse transcription Master Mix was added for cDNA synthesis. Reverse transcription Master Mix was removed after the end of first-strand synthesis, and 75 μL of 0.08 M KOH was added and incubated for 5 min at room temperature. Wells were washed with elution buffer, and 75 μL Second Strand Mix was added to each well for second-strand synthesis. cDNA amplification was performed on a thermal cycler. According to the manufacturer’s introduction, Visium spatial libraries were constructed using the Visium spatial Library construction kit (PN-1000184, 10× Genomics). The libraries were finally sequenced using an Illumina Novaseq 6000 sequencer with a sequencing depth of at least 100,000 reads per spot with a pair-end 150-bp reading strategy by LC-Bio Technology.

### ST Data Processing

Histology images and raw FASTQ files were processed using the Space Ranger pipeline (https://support.10xgenomics.com/spatial-gene-expression/software/pipelines/latest/what-is-space-ranger), and sequence data were aligned to the Ensembl genome GRCh38 reference genome. Published ST data of the TP myometrium was obtained from the GSA database (HRA002852) (30). The Space Ranger output was loaded into Seurat (version 4.0.2) to be used for dimensional reduction, clustering, and probabilistic transfer of cell populations from the scRNA-Seq data to the ST data (42, 43). Only keep spots that were determined to be over tissue and then 3,703 spots in NP and 4,285 spots in TP were obtained. Raw counts were normalized using SCTransform in Seurat. Deconvolution of ST spots was performed via SPOTlight as previously reported (44, 45). A total of *n* = 200 cells per cell type and *n* = 3,000 highly variable genes were used as input for the deconvolution.

### Bulk RNA-Seq Data Processing

The raw data and process data are available in GEO repositories, under Accession No. GSE137552 (27), which provides RNA-Seq results in human myometrial tissues from NP (*n* = 3) and TP (*n* = 3). DEGs in NP versus TP samples were identified using a two-sided Student’s *t* test through OmicShare tools (http://www.omicshare.com/tools), based on two criteria of a twofold change (FC) increase or decrease in expression levels, and a *P* value of <0.05.

### Gene Function Enrichment Analysis

Kyoto Encyclopedia of Genes and Genomes (KEGG) analysis of the gene list was performed using DAVID v6.8 online tools to annotate the signaling pathways (46). For each category, a *P* value of <0.05 was considered significant.

### Immunofluorescence Staining

Myometrial tissues were embedded in paraffin and sliced at 5 μm. Tissue sections were deparaffinized and rehydrated, followed by antigen retrieval in citrate buffer at 98°C for 10 min. After being blocked with 10% goat serum at 37°C for 1 h, sections were incubated overnight at 4°C with antibodies including anti-α-SMA (1:500, ab7817, Abcam) or anti-α-SMA (1:500, ab5694, Abcam), anti-VWF (1:500, ab6994, Abcam), anti-vimentin (1:200, ab8978, Abcam), CD163 (1:250, 16646-1-AP-100, Proteintech), CD68 (1:250, ab201340-500, Abcam), CD86 (1:100, ab269589, Abcam), and CK-7 (1:500, ab154334-100, Abcam). Sections were then incubated with Alexa Fluor 647 goat anti-mouse secondary antibody (1:500, ab15011, Abcam) or goat anti-rabbit Alexa Fluor 488-IgG antibody (1:500, ab150077, Abcam). Multiplex immunofluorescence was performed using the Goat Anti-Mouse/Rabbit Multiplex IHC Detection Kit (No. 18003, ZENBIO) and followed its protocol. Slides were counterstained with the nuclear dye DAPI and observed on a Leica DMi8 fluorescence microscopy.

### Immunohistochemistry Staining

Myometrial tissues were embedded in paraffin and sliced at 5 μm. After deparaffinization, tissue sections were treated with 3% hydrogen peroxide in methanol, blocked with a biotin-blocking kit (DAKO), and incubated overnight with CD68 (1:500, ab201340-500, Abcam), CD3(1:150, ab135372, Abcam), and CD66b (1:100, ab197678, Abcam) antibody in a humid chamber at 4°C. After being washed three times with PBS, slides were incubated with biotinylated goat anti-polyvalent antibodies for 1 h. Slides were stained with DAKO liquid 3,3′-diaminobenzidine tetrahydrochloride, followed by counterstaining with Mayer’s hematoxylin and examined under a microscope.

### Flow Cytometry

Fresh myometrial tissues were dissociated using single-cell preparation procedures. After digestion and washing, cells were resuspended in 100 μL Stain Buffer freshly prepared with 3 μL of anti-α-SMA (ab7817, Abcam). Cells were stained for 30 min on ice, followed by 1 μg of PE goat anti-mouse IgG antibody (No. 405307, BioLegend) for 20 min at 4°C. Flow cytometric analysis was performed on a BD LSRFortessa flow cytometer. Data analysis was performed using FlowJo software.

### Total RNA Isolation and Quantitative RT-PCR

Total RNA from myometrial tissues was extracted and purified using TRIzol reagent (No. 15596018, Invitrogen) according to the manufacturer’s instructions. At least 60 mg of myometrial tissues were homogenized in 1.5 mL of TRIzol reagent with an electric homogenizer and mixed with 0.3 mL of chloroform vigorously (Sigma Aldrich). After the mixture was centrifuged at 12,000 rpm for 10 min at 4°C, the RNA fraction was mixed with an equal volume of 100% ethanol. The mixture was centrifuged at 12,000 rpm for 20 min at 4°C, and the supernatant was then removed. After being washed with 75% ethanol, the RNA pellet was air-dried in the biosafety cabinet and then dissolved by DEPC-treated water. The RNA concentration was measured by Multiskan Go (Thermo Scientific). Total RNA was then reverse transcribed using the PrimeScript RT reagent Kit with gDNA Eraser (RR047A, TAKARA). Real-time PCR analysis was performed using the QuantStudio Real-Time PCR System (Applied Biosystems) with TB Green Premix Ex Taq II (Tli RNaseH Plus, RR820L, TAKARA). The sequence of primers used is provided in Supplemental Table S7. The PCR conditions were as follows: 95°C for 10 min, 40 cycles of 95°C for 15 s, and then 60°C for 1 min. Experiments were performed in triplicate samples and normalized with β-actin (ACTB); relative expression was calculated using the 2^−ΔΔCt^ method (where C_t_ is the threshold cycle).

### Statistical Analysis

The statistical tools, methods, and threshold for scRNA-Seq and ST data analysis are explicitly described with the results or detailed in the figures or materials and methods. Statistical differences between groups were assessed using a two-sided *t* test. *P* values of <0.05 were considered as statistically significant.

## RESULTS

### Single-Cell Transcriptome Atlas and Cell Typing in NP and TP Groups

**Figure 1.**
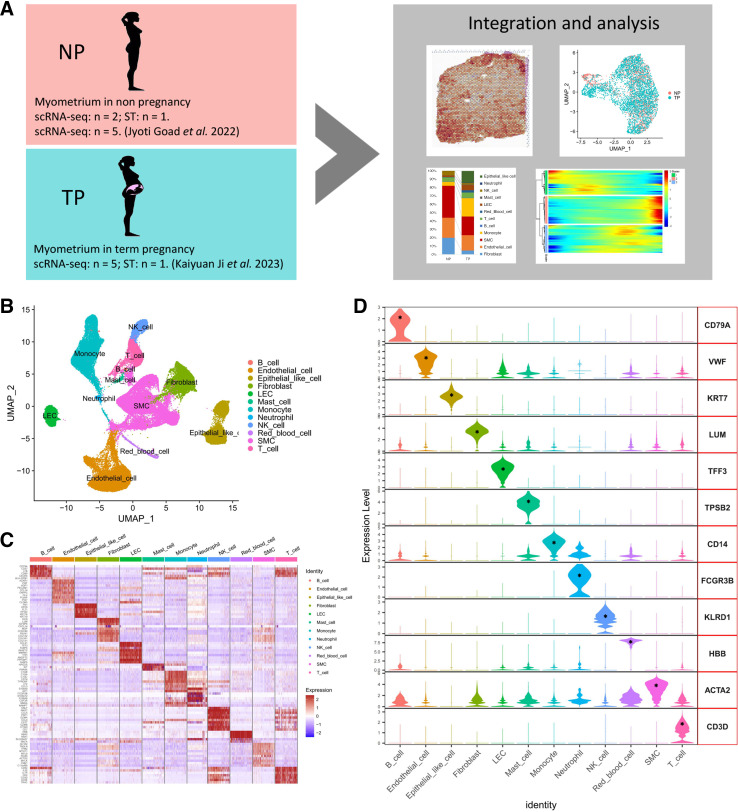
Diverse cell populations in the myometrium identified through scRNA-Seq analysis. *A*: workflow of scRNA-Seq and spatial transcriptomics in the human myometrium of NP and TP women. *B*: UMAP visualization of the major myometrial cell populations in scRNA-Seq of all NP and TP samples (NP: *n* = 7 and TP: *n* = 5). *C*: heatmap showing the relative expression of the top 10 genes in each cell population. *D*: violin plots showing the expression of canonical markers for each cell population. *P < 0.05. LECs, lymphatic endothelial cells; NP, nonpregnant; SMCs, smooth muscle cells; TP, term pregnant; UMAP, Uniform Manifold Approximation and Projection.

To understand the cellular diversity and molecular characteristics of the human myometrium in pregnancy and nonpregnancy, we collected scRNA-Seq and ST data from myometrial samples of both NP and pregnant individuals from databases and previous research (30, 36). In addition, we supplemented this with scRNA-Seq (*n* = 2) and ST (*n* = 1) data, and from the NP uterine myometrium and conducted integrated analyses (Fig. 1*A*). For scRNA-Seq, after an initial doublets were removed and a quality control step was performed (Fig. S1*A*), we processed the scRNA-Seq data using Seurat R packages for quality control, normalization, batch effect correction, data integration, and principal component analysis. Unsupervised UMAP clustering was used to partition the cells into clusters and visualize them. With the cell markers in previous studies and their sources (31, 47–49), 12 cell lineages were successfully identified among the 87,845 cells (32,099 in NP and 55,746 in TP) (Fig. 1*B* and Supplemental Table S2). In most cases, well-known cell type markers, such as LUM for fibroblasts, ACTA2 for SMCs, VWF for endothelial cells, CD79A for B cells, KRT7 for epithelial-like cells, TFF3 for lymphatic endothelial cells (LECs), TPSB2 for mast cells, FCGR3B for neutrophils, KLRD1 for NK cells, HBB for red blood cells, CD3D for T cells, and CD14 for monocytic cells, were used to determine cellular identity of the clusters (Fig. 1, *C* and *D*, Supplemental Fig. S1*C*, and Supplemental Table S3). The most abundant cell type was SMCs (28%), followed by endothelial cells (21%) and monocytic cells (16%) (Supplemental Fig. S1*B*), consistent with the known cellular composition of uterine tissues (28). We further confirmed the proportion of SMCs in the myometrium using flow cytometry, which was higher than that in the scRNA-Seq results (Supplemental Fig. S1*D*). To validate the presence of representative cell populations, we performed quantitative RT-PCR (Supplemental Fig. S2*A*) and immunofluorescence on myometrium samples of NP and TP women (Supplemental Fig. S2, *B–F*). Overall, we revealed the cellular composition of the myometrium and provided a comprehensive representation of myometrial cells for further respective studies during pregnancy.

### DEGs in the Myometrium Between NP and TP Women

**Figure 2.**
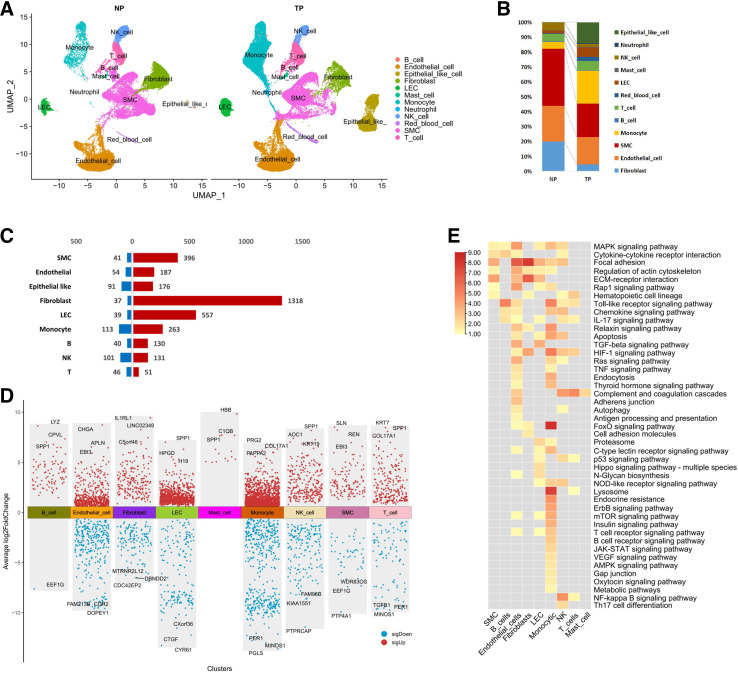
Differentially expressed genes (DEGs) of the myometrium between the NP and TP groups. *A*: UMAP visualization of myometrial cell populations in scRNA-Seq divided by groups. *B*: proportion of cell populations according to scRNA-Seq in the NP and TP groups. *C*: number of DEGs in each cell type based on scRNA-Seq with >100 cells available in the NP and TP samples through pseudobulk methods (significant DEGs were defined using an adjusted *P* value of < 0.05 and |fold change| of >1.5). *D*: volcano plot of the DEGs showing the top three upregulated and downregulated genes in major cell populations. Red represents upregulated and blue represents downregulated in the TP group. Genes with the prefix “MT” have been filtered out. *E*: heatmap showing the representative KEGG pathways enriched in DEGs in each cell population. KEGG, Kyoto Encyclopedia of Genes and Genomes; NP, nonpregnant; TP, term pregnant; UMAP, Uniform Manifold Approximation and Projection.

To define gene expression changes at the global and cellular levels simultaneously, we analyzed bulk RNA-Seq data of myometrium samples from NP and TP women in the GSE137552 datasets. In the bulk RNA-Seq data, 356 upregulated genes and 390 downregulated genes were detected in the TP group (Supplemental Fig. S2*G* and Supplemental Table S4). GO analyses were performed on these genes (Supplemental Fig. S2*H* and Supplemental Table S5), indicating that extracellular exosome, ECM organization, cell adhesion, platelet degranulation, and angiogenesis were activated specifically in the TP group. The downregulated genes in the TP group were enriched with regard to collagen trimer, ion, and chloride transmembrane transport. To explore specific changes in the expression of molecules in each cell type during labor, we analyzed the proportions of cell lineages in scRNA-Seq data from NP and TP groups. The cell lineages of the NP and TP myometrium differed in cell number ratios (Fig. 2, *A* and *B*). Increased proportions of monocytic cells were observed in the TP group. The proportions of SMCs and fibroblasts were decreased in the TP group, which may be due to the expansion of these cells in TP tissues, and some of them could not pass the filter before getting into the single-cell systems. We then counted DEGs using the pseudobulk method between the two groups in the main cell types (Supplemental Table S6), and numerous DEGs occurred in multiple cell types (Fig. 2, *C* and *D*). KEGG enrichment analysis of DEGs for each cell type indicated that the altered pathways are primarily enriched in immune and ECM-related pathways (Fig. 2*E*).

### Integration of scRNA-Seq and ST in NP and TP

**Figure 3.**
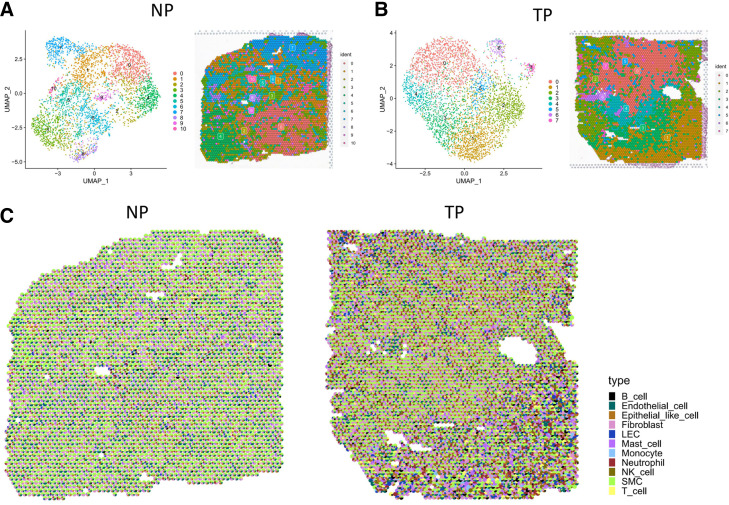
Spatial transcriptomics of the myometrium in the NP and TP groups. *A* and *B*: UMAP and spatial feature plots of clustered ST in the NP and TP groups. *C*: spot prediction pie chart of ST data of NP and TP groups. Each small pie chart represents a spot, filled with colors representing different cell types. NP, nonpregnant; ST, spatial transcriptomics; TP, term pregnant; UMAP, Uniform Manifold Approximation and Projection.

To visualize the cell populations and spatial distribution of the myometrium, we analyzed ST data on the myometrium in NP and TP samples (*n* = 1, each). After filtering, transcriptomes from 3,703 spots and 4,285 spots in the NP and TP groups were obtained, respectively. A correlation coefficient of 0.98 was observed between the genes and UMIs in NP and TP; the counts per spot are shown in Supplemental Fig. S3*A*. To show the spatial organization of cell populations in an unbiased manner, we performed deconvolution analysis for each spot with cell type signature genes derived from scRNA-Seq using SPOTlight. After deconvolution, we found that SMCs had the highest enrichment score in most spots, indicating that they are the predominated cell components of the myometrium, whereas the enormous size of SMCs cannot be ruled out either (Fig. 3*C*). Some spots were highly enriched in endothelial cells and fibroblasts. The number of endothelial cell spots was more than that of the NP myometrium, and fibroblasts showed no difference between the two groups. Few spots were matched to immune cells, especially in the NP myometrium. The expression of the marker genes on each spot of spatial transcriptomics is provided in Supplemental Fig. S3*B*.

### Determination of Differential Cell-Cell Communication Patterns Between NP and TP Women

**Figure 4.**
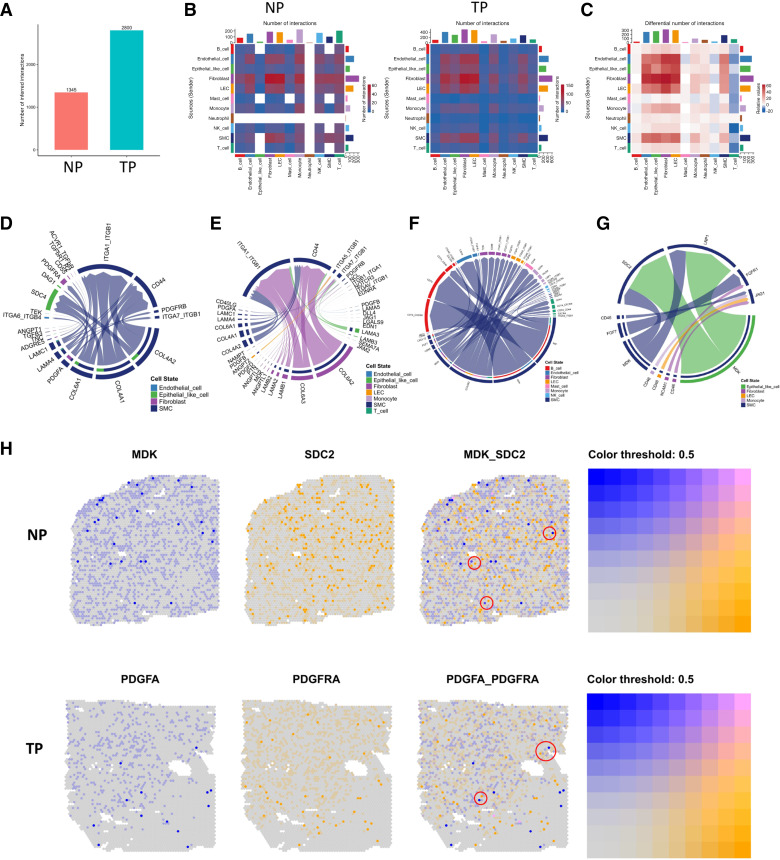
Communications between distinct cell populations. *A*: interaction numbers among each cell type in the NP and TP groups. *B*: heatmap showing the number of interactions between each cell type. Bars shown on the *top* and *right* side indicate the interaction number between two populations (sender and receiver). *C*: comparison of the differential number of interactions between each cell type. The number represents increased signaling pairs in the TP group compared with the NP group. Bars shown on the *top* and *right* side indicate the interaction number between two populations (sender and receiver). *D–G*: circle plot showing the cellular interaction of SMCs with other cell populations. *D*: upregulated cellular interaction in the TP group, with SMCs as the source. *E*: upregulated cellular interaction in the TP group, with SMCs as the target. *F*: downregulated cellular interaction in the TP group, with SMCs as the source. *G*: downregulated cellular interaction in the TP group, with SMCs as the target. *H*: spatial distribution of representative differentially expressed ligands and receptors (MDK-NCL and PDGFA-PDGFRA) in ST data of the myometrium from the NP and TP groups. Circles indicate the adjacent expressed ligands and their receptors. NP, nonpregnant; SMCs, smooth muscle cells; ST, spatial transcriptomics; TP, term pregnant.

To define the intercellular communication networks within the myometrium in both groups, we performed analyses using the R package CellChat. Our results showed that the interaction number increased in the TP group (Fig. 4*A*). The total number of interactions between cell clusters is shown in Fig. 4*B*. We calculated the differential interactions of each major cell population between NP and TP, and the number of interactions in each cell populations was increased in the TP group (Fig. 4*C*). The major changes were attributable to SMCs, endothelial cells, fibroblasts, and LECs. Specifically, we analyzed the upregulated and downregulated interactions between SMCs with other major cell populations in the TP group when SMCs acted as the source/target. The results showed that PDGFA-PDGFRA, PDGFA-PDGFRB, COL4A1-ITGA11_ITGA1/CD44, and LAMA3-CD44 were upregulated in the TP group when SMCs were the source of communication with other cells (Fig. 4*D*). PDGFA signals participate in angiogenesis and are associated with the cGMP pathway (50, 51). Collagen-related interactions (COL1A2, COL6A1, COL6A2, and COL6A3) were upregulated in TP samples when SMCs were the target (Fig. 4*E*). These results indicate the participation of SMC and fibroblast populations in the collagen signaling network during myometrial remodeling in pregnancy. Mainly, APP and MIF-related interactions were downregulated when SMCs were the source (Fig. 4*F*). MDK-SDC2/LRP1 interactions were downregulated when SMCs were the target (Fig. 4*G*), which was associated with immune cell activation (52). We also showed expression of the ligand-receptor interaction of MDK-SDC2 and PDGFA-PDGFRA in ST results to indicate their spatial proximity (Fig. 4*H*).

### Identification and Characterization of SMC Subpopulations in the Myometrium

**Figure 5.**
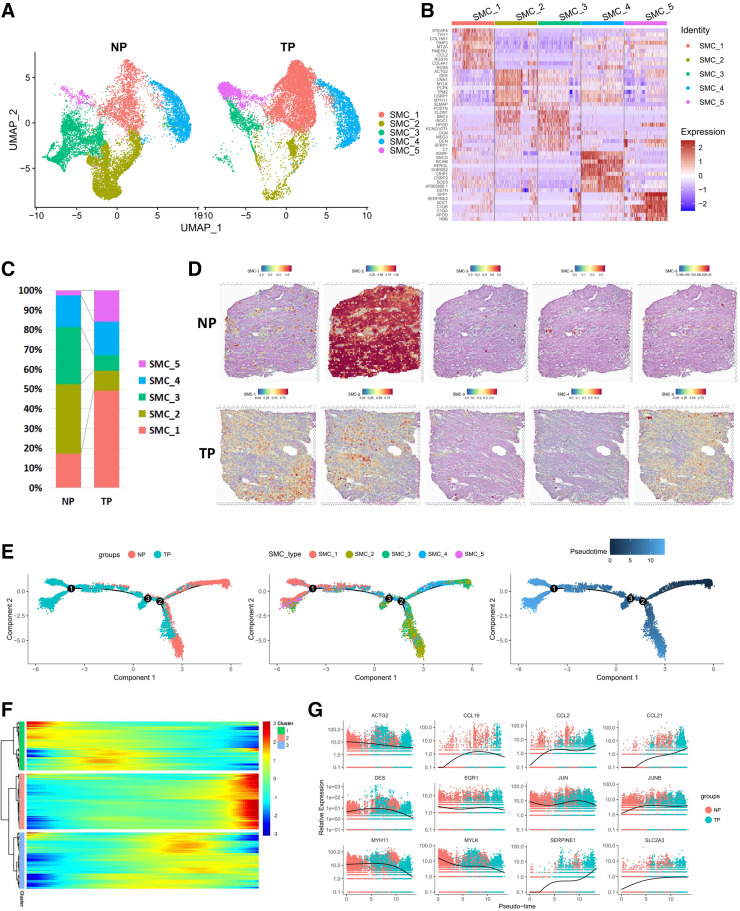
SMCs of NP and TP samples subcluster into distinct cell populations. *A*: UMAP visualization of SMC subpopulations divided by groups. *B*: heatmap showing the relative expression of the top 10 genes in each SMC subpopulation. *C*: proportion of SMC subpopulations in scRNA-Seq in the NP and TP groups. *D*: SMC subpopulations mapping onto spatial transcriptomics. *E*: pseudotime analysis depicting the developmental trajectory of SMCs from NP to TP. *F*: three distinct groups of pseudotime-dependent genes with dynamic expression patterns plotted across pseudotime as heatmaps, with blue indicating low levels and red indicating high levels of expression. *G*: scatterplots showing the expression change of selected marker genes in NP and TP along the pseudotime trajectory. NP, nonpregnant; SMCs, smooth muscle cells; TP, term pregnant; UMAP, Uniform Manifold Approximation and Projection.

From the UMAP results shown in Fig. 1*B*, we observed that SMC populations produced more than one cluster. As is widely known, SMCs underwent significant changes during pregnancy. We performed unsupervised clustering on all SMC cells in NP and TP samples and identified five distinct SMC clusters in the myometrium (Fig. 5*A*). Figure 5*B* shows the top 10 DEGs of each subpopulation. The SMC-1 subpopulation expressed STEAP4, THY1, and COL18A1. The SMC-2 subpopulation was highly expressed in DES and ACTG2. SMC-3 was highly expressed in CLDN1 and BNC2. SMC-4 was highly expressed in ADIRF and SNCG. SMC-5 was highly expressed in DSTN and SPP. The proportions of the SMC subpopulations showed significant differences between NP and TP samples (Fig. 5, *A* and *C*). In the TP group, the proportions of SMC-2 and SMC-3 were decreased, whereas SMC-1 and SMC-5 were increased. We mapped five subpopulations into ST data using “FindTransferAnchors” function. The results indicated that in the ST data of the NP myometrium, SMC-2 was the predominant cell type, covering the majority of the area. In contrast, the ST data of the TP myometrium showed a dominant presence of SMC-1, SMC-2, and SMC-5. The expression of canonical contraction-related genes in SMC subpopulations is provided in Supplemental Fig. S4*A*, cells that highly expressed GJA1 belonged to the SMC-5 cluster. PTGFR was mainly expressed in SMC-2. OXTR was highly expressed in SMC-2 of TP group. However, few SMCs expressed PTGS2 and IFNG. To understand the hypothetical evolution trajectory that might exist within SMCs, pseudotime analysis was performed by Monocle, which orders SMCs in an unsupervised manner by examining the pattern of gene expression, revealing the three branches and five subpopulations (Fig. 5*E*). The genes that contributed to the pseudotime could be clustered into three patterns (Fig. 5*F*). Cytoskeleton protein ACTG2, DES, MYH11, and MYLK were downregulated during pseudotime (Fig. 5*G*). CCL2, CCL21, SCL2A3, and SERPINE1 were upregulated during pseudotime. The expression of CCL19, EGR1, and JUN initially increased and then decreased. These results revealed the differential expression of genes from NP to TP at the single-cell level, thus highlighting significant changes in gene expression during myometrium remodeling.

### Monocytic Cells Are Increased in the TP Group

**Figure 6.**
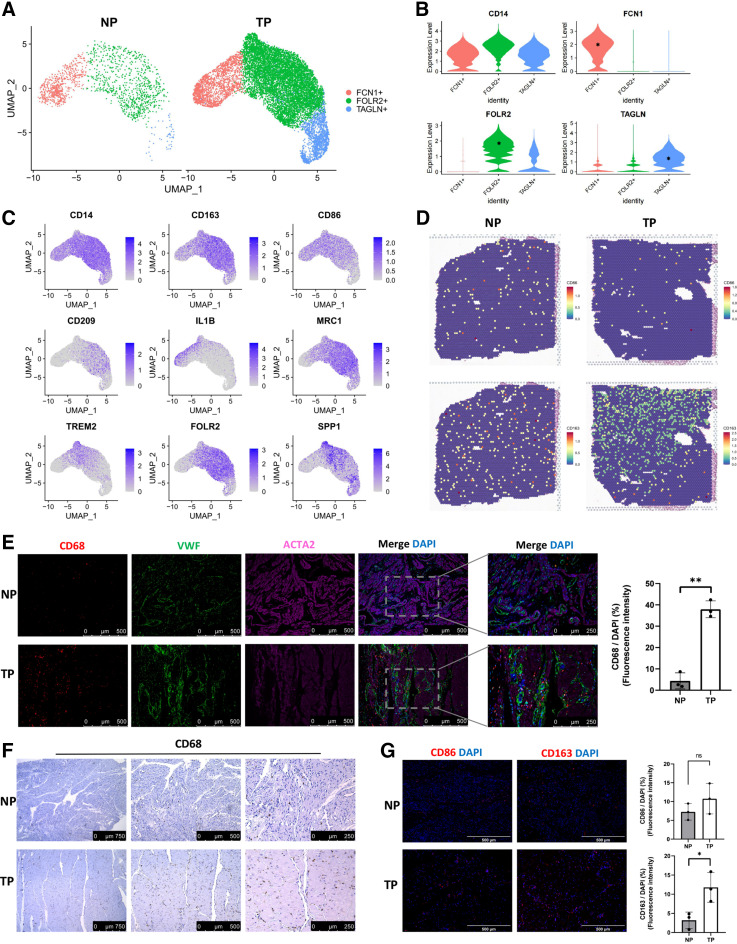
Distinct subgroups of monocytes in the NP and TP samples. *A*: UMAP visualization of monocytic subpopulations divided by groups. *B*: violin plots representing the marker genes of each monocytic subpopulation. *P < 0.05. *C*: UMAPs color-coded for expression (gray to blue) of canonical monocytic markers in monocytic subpopulations. *D*: spatial plots showing monocytic markers for M1 and M2 monocytic subpopulations. *E*: immunofluorescence staining of CD68 and VWF in the NP and TP myometrium. Also shown is the quantification of CD68 staining (*n* = 3 women for each group). Data are presented as means ± SD. **P < 0.01. *F*: immunohistochemical staining of CD68 in the NP and TP myometrium. *G*: immunofluorescence staining and quantification of CD86 and CD163 in the NP and TP myometrium (*n* = 3 women for each group). Data are presented as means ± SD. **P < 0.01. NP, nonpregnant; TP, term pregnant; UMAP, Uniform Manifold Approximation and Projection.

scRNA-Seq showed that the cell population of monocytic cells increased markedly in the TP group. Using unsupervised clustering and identification of monocytic cell markers, we identified three monocytic subpopulations (FCN1+, FOLR2+, and TAGLN+) (Fig. 6, *A* and *B*). FCN1+ monocytes expressed high levels of FCN1 and CD14, and part of them expressed IL1B. FOLR2 is a marker of tissue-resident macrophages, expressed by subpopulations of FOLR2+ and TAGLN+. FOLR2+ monocytes exhibit higher expression levels of FOLR2. Most of the monocytes strongly expressed CD163 and MRC1 (CD206), which is the main phenotype of M2 macrophages (Fig. 6*C*). Some cells coexpressed TREM2, SPP1, and FOLR2, indicating no clear exclusivity (53). Unexpectedly, the classic macrophage marker CD68 expressed low in scRNA-Seq data. To explore the change of inflammation, we analyzed ST data, and results showed more spots expressing the M2 marker (CD163) in the TP sample (Fig. 6*D*). Histological identification showed that numerous macrophages (CD68) occurred in the myometrium of TP women (Fig. 6, *E* and *F*), and some macrophages infiltrated into the gaps between SMCs in the TP myometrium, but the macrophages in NP myometrium did not. Immunofluorescence results of the NP and TP myometrium confirmed significantly higher infiltration of M2 (CD163) macrophages in TP samples (Fig. 6*H*). As for the immunosuppressive marker, monocytic cells of the TP group expressed higher HAVCR2, IL10, and IL2RA than the NP group (Supplemental Fig. S4*B*).

### Distribution of Lymphocytes in the Myometrium

**Figure 7.**
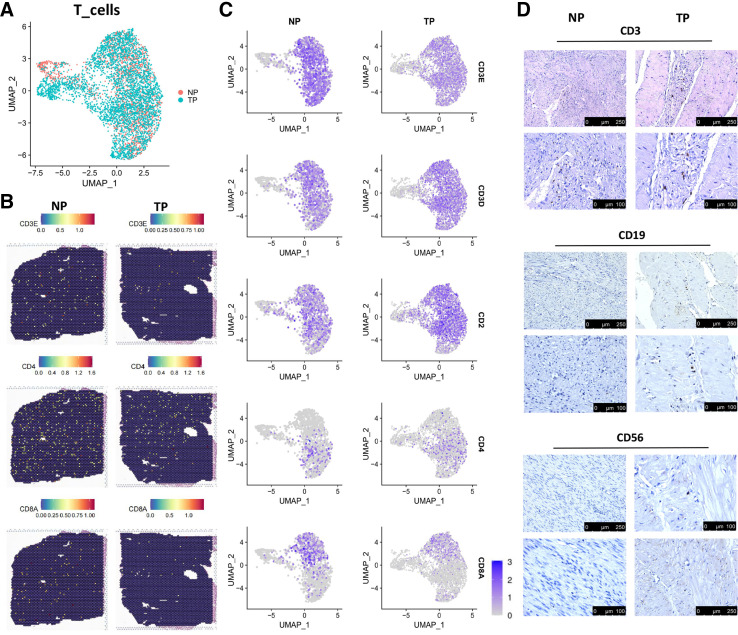
Lymphocytes in the NP and TP groups. *A*: UMAP visualization of T cells in the NP and TP groups. *B*: spatial plots showing canonical T cell markers. *C*: UMAPs color-coded for expression (gray to blue) of canonical T cell markers in the NP and TP groups. *D*: immunohistochemical staining of CD3, CD19, and CD56 in the NP and TP myometrium. NP, nonpregnant; TP, term pregnant; UMAP, Uniform Manifold Approximation and Projection.

T cells accounted for a large proportion according to the scRNA-Seq data. We further identified T cell subpopulations to investigate their transition in TP. However, CD4+ and CD8A+ T cell abundances showed no difference between NP and TP groups in scRNA-Seq and ST data (Fig. 7, *A*–*C*). We performed immunochemical staining to display the T cells distribution in myometrium, showing that T cells could infiltrate into the myometrium of NP and TP women (Fig. 7*D*). To explore the immunosuppressive microenvironment in the myometrium, we analyzed the expression of canonical immune tolerance markers in T cells; however, few T cells expressed CD27 and FOXP3 (Supplemental Fig. S4*B*). Moreover, immunochemical staining of CD56 and CD19 indicated few NK and B cells in the myometrium (Fig. 7*D*).

### Heterogeneity of Fibroblasts, Endothelial Cells, and Epithelial-Like Cells in NP and TP Samples

**Figure 8.**
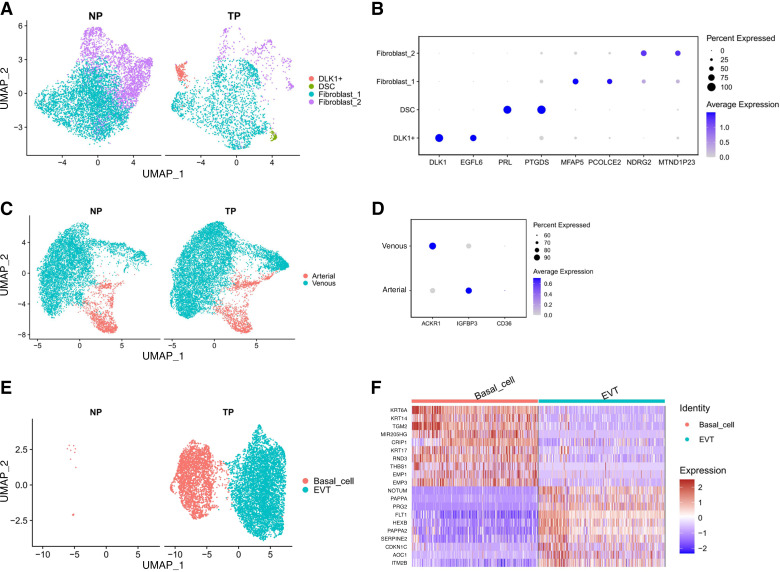
Distinct subpopulations of fibroblasts, endothelial cells, and epithelial-like cells in the NP and TP samples. *A*: UMAP visualization of fibroblast subpopulations divided by groups. *B*: dot plot showing the relative expression of marker genes in each fibroblast subpopulation. *C*: UMAP visualization of endothelial subpopulations divided by groups. *D*: dot plot showing canonical marker genes of arterial, venous, and capillary endothelial cells in each endothelial subpopulation. *E*: UMAP visualization of epithelial-like subpopulations divided by groups. *F*: heatmap showing the relative expression of the top 10 genes in each epithelial subpopulation. NP, nonpregnant; TP, term pregnant; UMAP, Uniform Manifold Approximation and Projection.

Fibroblasts are crucial for maintaining extracellular integrity under mechanical stretching and regulating muscle contraction (54, 55). We further identified the cell populations of the fibroblast cluster in the scRNA-Seq data. Fibroblasts were heterogeneous and clustered in four groups: DLK1+, decidual stromal cells (DSCs), and two clusters of fibroblasts (fibroblast-1 and fibroblast-2) (Fig. 8, *A* and *B*). DLK1 is predominantly expressed from the paternally inherited chromosome during fetal development (56). DLK1+ fibroblasts probably were fetal cells that attach or implant to the myometrium of TP. Similar to DLK1+ cells, DSCs only exist in TP groups, which is due to the fact that during sample collection and decidua detachment, a small number of them remained adhered to the tissues. Fibroblast-1 and fibroblast-2, respectively, highly expressed MFAP5 and NDRG2. MFAP5 is a fibroblast-derived mediator and plays a significant role in wound healing, and NDRG2 is reported to inhibit fibrosis (57, 58). Obviously, fibroblast-2 decreased in the TP myometrium. Endothelial cells could be identified as arterial and venous endothelial cells (Fig. 8*C*). Capillary vessel endothelial cells (CD36+) were not found in the data (Fig. 8*D*). Epithelial-like cells were unexpectedly detected in our analysis. Based on the marker of two clusters of them, we divided them into basal cells (uterine epithelia) and extravillous trophoblasts (EVTs), which highly expressed KRT6A and PAPPA, respectively (59–61).

## DISCUSSION

The myometrium plays an important role during pregnancy as it is required for the adequate nourishment and development of the fetus. To accommodate fetal growth, the myometrium undergoes extensive remodeling in structure and function. It is thus important to explore the myometrial remodeling during pregnancy. Herein, we performed single-cell and spatiotemporal transcriptome analyses to reveal the various cell populations in the NP and TP myometrium. Notably, 12 main cell populations, including 5 SMC subpopulations and immune cell subpopulations, were identified. DEGs and biological functions between NP and TP in the major cell populations were defined, and cell-cell communication patterns were described. Moreover, we identified alterations in the subpopulations of SMC, fibroblasts, and monocytic cells during pregnancy. These findings will improve our understanding of myometrial remodeling during pregnancy in depth and provide potential targets for clinical therapy of preterm birth.

The cell populations differed between TP and NP samples, whereas the major cell populations were the same. SMCs, endothelial cells, fibroblasts, and immune cells are the major cell populations in the myometrium, accounting for over 90% of all cells. Important biological processes linked with myometrial remodeling during pregnancy, such as extracellular matrix organization and angiogenesis, have been revealed via bulk RNA-Seq; however, these results may not explain whether these changes are intrinsic molecular changes or simply reflect changes in the proportions of the cell populations (27, 48). The array of ST contained 5,000 spots in a capture area (6.5 × 6.5 mm). The diameter of each spot was 55 µm, so there were 1–10 cells on average on each spot. The various cell types in the myometrium show different sizes, including large SMCs, which can cover one or two sites in a capture area (28). In our ST data, most spots were mapped to SMCs. The proportion of SMCs in the section closely matched that in the histological staining and flow cytometry (Fig. 3 and Supplemental Fig. S1*D*). The size of SMCs was large (50–100 µm) (28), which could cover more than one spot, and thus might have interfered with the identification of other smaller cells, such as immune cells. The number of samples for ST is a limitation in our study. Meanwhile, we performed histological staining to supplement the results of scRNA-Seq and ST. Even so, integration of scRNA-Seq and ST data may be most suitable to reveal the heterogeneity in myometrial cell populations and spatial gene expression patterns at present (62, 63). Leiomyomas are more common in women between the ages of 30 and 50 yr (64, 65). The patients of the NP group were older than those of the TP group in our data, which is a limitation of our research. However, it was difficult to obtain ideal clinical samples, and we recruited samples from patients who were still of reproductive age, whose hormone levels, follicle-stimulating hormone (FSH), luteinizing hormone (LH), anti-Müllerian hormone (AMH) were in the normal range of women of normal reproductive age.

SMCs comprised the major cell population in the myometrium, accounting for 28.05% of scRNA-Seq data. We obtained the percentage of the SMC decrease in TP samples, considering hypertrophy of SMCs in TP tissue leading to the decrease of cell number in the same volume; some SMCs were too large to pass the filter before getting into the single-cell system. However, the spatiotemporal transcriptome showed that most spots were mapped to the SMC and highly expressed its markers, at a higher proportion than in the scRNA-Seq data. Flow cytometry was performed to verify α-SMA (SMA+) cell populations and showed the proportion of SMCs in a further aspect. We suggest that the cell number proportion of each cell population as reflected in the scRNA-Seq data more accurate. The spatiotemporal transcriptome preferably shows the area of cell populations and is susceptible to the location of section and the area of cells. Deconvolution of the spots indicated that multiple cell populations existed in one spot. approaches, like single-nuclei RNA-seq (snRNA-Seq), which uses isolated nuclei instead of the entire cells, can avoid the issue of oversized SMC. But, snRNA-Seq will lose some mRNA from the cytoplasm and is unfriendly to immune cells (66, 67). However, scRNA-Seq and spatiotemporal transcriptome data indicated that SMCs significantly changed during pregnancy. According to our results and the results of previous studies (48, 68), we suggest that SMC-2 and SMC-3 may transform into SMC-1 and SMC-5 during pregnancy. In TP tissue, maintaining the quiescence of SMCs is a prerequisite throughout pregnancy, which is caused by a delicate balance between hormonal, inflammatory, and other physical factors (69, 70). Increasing progesterone levels and mechanical stretching are the most pronounced characteristics during pregnancy. Under treatment with medroxyprogesterone acetate, DEGs of myometrial explants are significantly enriched with regard to the inflammatory response, growth factor activity, and cytokine activity genes (71).

Immune cells can be recruited into the reproductive tract (cervix and myometrium) and decidual tissues during pregnancy (22). Our scRNA-Seq data confirmed this and reflected the alterations of each subpopulation of monocytic and T cells during pregnancy. Macrophages increased extremely in the TP group, and most of them were M2 (highly expressed CD163). The enrichment of M2 macrophages could decrease inflammation and encourage myometrium repair in the TP myometrium, thus playing a crucial role in maintaining quiescence of the myometrium and avoiding immunological rejection. A previous study indicated that macrophages exert homeostatic actions during pregnancy to protect against preterm birth and fetal inflammatory injury in late gestation. M2 macrophages showed a superior capacity over nonpolarized macrophages to reduce uterine and fetal inflammation, prevent preterm birth, and improve neonatal survival (23). In a model of intra-amniotic inflammation-induced preterm birth, macrophages polarized in vitro to an M2 phenotype could reduce uterine and fetal inflammation, prevent preterm birth, and improve neonatal survival. In mice, systemic macrophage depletion causes fetal morbidity and mortality (23).

Hormones and tension play vital roles in myometrial physiology and contractility during pregnancy (72, 73). Following medroxyprogesterone acetate treatment, expression of C1QB and CD163 increased in myometrial explants (74) and IL-6, IL-11, and IL-1β decreased (71). Progesterone can suppress T cell activation to attenuate proinflammatory responses at the maternal-fetal interface (75, 76). At the end of TP, the myometrium is maximally stretched to accommodate the growing fetus, which may also affect the cell populations in the myometrium. Under tension, expression of the chemokines CXCL6 and CCL21 is upregulated and that of IL-23A is downregulated in the myometrium (13). Our results showed that few cells expressed markers of regulatory T cells, such as CD25 (IL2RA) and FOXP3. We attempted to find further evidence of immunosuppression such as PD-L1 (CD274), HAVCR2, CTLA4, and EBI3. We found that CD274 and EBI3 in EVT, HAVCR2, and IL2RA in monocytes and LAG3 in NK cells likely play significant roles in immunosuppression at the uterine-myometrial interface during late pregnancy. Our previous study also identified that supplementation with choline protects against gestational lipopolysaccharide-induced inflammatory responses during pregnancy (77). We reached a deeper understanding of the innate and adaptive immune cell components involved in pregnancy, which may help guide us to develop strategies to prolong pregnancy and thereby improve pregnancy outcomes. Interestingly, a small group of cells in the TP myometrium strongly expressed PAPPA and KRT7, suggesting that these cells are trophoblast cells. Generally, trophoblast cells do not occur in the myometrium, which is collected from the lower uterine segment. Similar results were observed through scRNA-Seq of the myometrium in labor (28). We will further explore the functions of these unexpected cell types in subsequent studies.

Cell-cell communication is important for various biological processes. Our results showed that intercellular communication increased in the TP group. As the largest component in the myometrium, SMCs play a core role in intercellular communication. PDGFA and collagen signals were upregulated, which participate in angiogenesis, and this indicated collagen signaling remodeling during pregnancy. The interaction of FN1 and MDK-SDC2/ITGAV_ITGB1 was downregulated, which is associated with immune cell activation. The exact mechanisms of how these signaling pathways are synergistically regulated by a variety of distinct cells and function stably warrant further research.

In summary, the single-cell and ST atlases of the human myometrium showed cellular heterogeneity, which broadened our understanding of cell identities and their spatial location in the myometrium during pregnancy. We present the alteration of five SMC subpopulations in the NP and TP groups. In addition, we identified an increase in M2 macrophages in the TP group, suggesting that they were essential for the anti-inflammatory response and immune tolerance during pregnancy. These findings will help improve our understanding of these cells and their roles in pregnancy, and our study provides important data support for research on abnormal pregnancy and preterm labor.

## DATA AVAILABILITY

Raw sequencing data and processed data are available through the Genome Sequence Archive (https://ngdc.cncb.ac.cn/gsa/) under Accession No. HRA002852. The code to reproduce the analyses described in this study can be accessed via https://github.com/feitianerguotou/np_tp. All other data supporting the findings of this study are available within the article and its Supplemental Data files or from the corresponding authors upon reasonable request. Source data are provided in this paper.

## SUPPLEMENTAL DATA

10.6084/m9.figshare.24418870.v1 Supplemental Figs. S1–S4: https://doi.org/10.6084/m9.figshare.24418870.v1.

10.6084/m9.figshare.24321577.v1 Supplemental Tables S1–S7: https://doi.org/10.6084/m9.figshare.24321577.v1.

## GRANTS

This work was supported by the National Natural Science Foundation of China (Grant 81871181), the Foundation of Guangzhou Municipal Science and Technology Bureau (Grant 202102010016), and the High-Tech Major Featured Technology Program of Guangzhou Municipal Health Commission (Grant 2019GX07).

## DISCLOSURES

No conflicts of interest, financial or otherwise, are declared by the authors.

## AUTHOR CONTRIBUTIONS

K.J., J.Z., L.C., and H.L. conceived and designed research; K.J., L.C., X.W., L.-N.C., B.W., F.Y., W.D., X.P., L.W., J.B., and Y.C. performed experiments; K.J. and L.C. analyzed data; K.J. and L.C. interpreted results of experiments; K.J. and L.C. prepared figures; K.J. and L.C. drafted manuscript; K.J., J.Z., L.C., and H.L. edited and revised manuscript; K.J. and H.L. approved final version of manuscript.
